# Detection of Antilisterial Activity of 3-Phenyllactic Acid Using *Listeria innocua* as a Model

**DOI:** 10.3389/fmicb.2018.01373

**Published:** 2018-06-26

**Authors:** Elena Sorrentino, Patrizio Tremonte, Mariantonietta Succi, Massimo Iorizzo, Gianfranco Pannella, Silvia Jane Lombardi, Marina Sturchio, Raffaele Coppola

**Affiliations:** Department of Agricultural Environmental and Food Sciences, University of Molise, Campobasso, Italy

**Keywords:** PLA, *Listeria innocua*, antimicrobial activity, phenolic compounds, biopreservation

## Abstract

The 3-Phenyllactic acid (PLA) produced by various lactic acid bacteria (LAB) possesses a broad spectrum of antimicrobial activity. In this study, the effect of PLA against *Listeria innocua* was studied with the aim to obtain additional information about its mechanism of action. The effect of pH on the antilisterial activity of PLA was investigated and a pH-dependent behavior, typical of weak acid, was detected. The antilisterial effect of PLA was firstly compared to that produced by lactic acid (LA) and than to that expressed by phenolic acids (gallic, caffeic, and ferulic acids) evaluating minimum inhibitory concentration (MIC), MBC, and survival kinetic parameters. PLA showed MIC values and death kinetic parameters significantly different from those exhibited by LA and by tested phenolic acids. In particular, the MIC value observed for PLA *vs L. innocua* resulted lower than that of the other preservative compounds studied herein, and consistent with the quantity generally produced by LAB. Moreover, the effect of PLA and phenolic acids on bacterial surface charge and loss of cellular content resulted different. The overall results highlighted strong differences in the antilisterial mechanism of action among PLA and other compounds such as LA and phenols. Specifically, it is possible to hypothesize that the antilisterial mechanism of action due to PLA is associated with the affinity to cell surface, which contributes to the cellular damage.

## Introduction

In the last years, several bio-control strategies have been developed to improve the safety and to extend the shelf-life of various foods. In this field, protective microbial cultures (Sorrentino et al., 2013; Tremonte et al., 2017a) and treatments with natural substances (Reale et al., 2011; Tipaldi et al., 2011; Catalano et al., 2015; Tremonte et al., 2016a) showed positive effects. The use of natural compounds, in particular antimicrobials produced by protective cultures, may represent an important tool in the food biopreservation, also considering the consumer demand for safe and healthy foods, in terms of reduction in chemical additives and provision of beneficial effects (Reale et al., 2011; Succi et al., 2014). In this context, 3-phenyllactic acid [2-Hydroxy-3-Phenyl propionic acid (PLA)], an organic acid produced by several microorganisms used in the food industry, shows promising results. In fact, some Authors highlighted that PLA has antimicrobial activity against fungi and bacteria (Mu et al., 2012; Liu et al., 2017; Ning et al., 2017), and it could be used as natural bio-control agent to extend the shelf-life of foods (Lavermicocca et al., 2003; Schnürer and Magnusson, 2005; Schwenninger et al., 2008). In a recent review by Chaudhari and Gokhale (2016), the production of PLA by various microorganisms, including metabolic pathways, and the methods applied for its detection were described. Interestingly, PLA can be produced by many microorganisms, especially lactic acid (LA) bacteria (Schnürer and Magnusson, 2005; Coloretti et al., 2007; Schwenninger et al., 2008; Mu et al., 2009). However, the information regarding the mechanism of action is still under investigation, even if some authors supposed that PLA damages the cell wall or the cytoplasmic membrane when bacteria are exposed to it (Dieuleveux et al., 1998; Ning et al., 2017). Recently, PLA has been reported to be able to produce promising antilisterial activity (Liu et al., 2017; Ning et al., 2017). Actually, *Listeria monocytogenes* is a major human foodborne pathogen, and it is one of the most deleterious bacteria in the food industry. Although listeriosis is relatively rare, this disease is severe, especially for vulnerable individuals such as pregnant women, elderly, newborns and immune-compromised patients, with symptoms ranging from septicemia, meningitis, encephalitis, and abortion to death (Drevets and Bronze, 2008; Hilliard et al., 2018). Many reports have demonstrated the interaction between *L. monocytogenes* and food matrices, showing the high adaptive capacity of this bacterium to survive in extreme environmental conditions. For example, environmental sub-lethal pH may induce resistance mechanisms to acid stress, which make the *Listeria* cells more resistant to severe acid conditions (Gandhi and Chikindas, 2007).

On the basis of these evidences, the individuation of new antilisterial compounds and the investigation of their mode of action represent a crucial step for the preservation of food safety. To date, antimicrobial activity of PLA, including antilisterial ability, was already recognized, but, as stated above, the antimicrobial mechanism of PLA is still not clarified. Ning et al. (2017) supposed that PLA has a mode of action different to that of other organic acids, such as LA, and this fact is due to the PLA chemical structure, showing amphiphilic properties resulting from the hydrophobic group-benzene ring, and hydrophilic group-carboxy group. For this reason, PLA could be able to interact with both lipids and proteins in the cell membrane, and hence to affect its permeability and integrity, with an antimicrobial mechanism similar to that highlighted in the case of other phenolic acids.

Despite its positive antimicrobial effect, PLA, as a pure compound, is still not included in the list of GRAS (*Generally Recognized As Safe*) additives (Chaudhari and Gokhale, 2016). However, in the last years, great attention has been focused on bacterial strains able to produce PLA, due to their broad-ranging antimicrobial activity.

In the light of previous findings, the present study aimed at the evaluation of the antilisterial mechanism of PLA at different pH values, choosing a concentration of the organic acid compatible with that normally found in LAB cultures (Rodríguez et al., 2012; Cortés-Zavaleta et al., 2014; Corsetti et al., 2015). Moreover, the antilisterial activity of PLA was compared to that obtained with the use of LA and phenolic compounds, since the antibacterial activity is not only dependent on the pH produced by organic acids, but it is also related to their chemical structures (Hismiogullari et al., 2008).

For this purpose, *Listeria innocua* was used as a non-pathogenic surrogate for *Listeria monocytogenes* considering the high similarity between the two species (Tremonte et al., 2016b).

## Materials and Methods

### Chemicals

The 3-Phenyllactic acid (PLA), lactic acid (LA), gallic acid (GA, 3,4,5-trihydroxybenzoic acid), ferulic acid (FA, 3-methoxy-4-hydroxycinnamic acid), and caffeic acid (CA, 3,4-dihydroxycinnamic acid) were purchased from Sigma-Aldrich (St. Louis, MO, United States). For each acid, a stock solution was prepared by dissolving an appropriate amount in dimethyl sulfoxide (DMSO, Sigma-Aldrich) at a final concentration of 100 mg/mL. Stock solutions were sterilized by filtration (0.22 μm pore-size membrane; Millipore, Bedford, MA, United States) and stored at 4°C before use.

### Bacterial Strain and Growth Media

*Listeria innocua* ATCC 33090 obtained from the Leibniz Institute DSMZ-German Collection of Microorganisms and Cell cultures was maintained in cryovials at -80°C and propagated twice in Brain Heart Infusion medium (BHI, Oxoid, Milan, Italy) at 37°C. Prior to use, cells were revitalized twice in the same medium and incubation conditions, and collected in the middle of exponential phase.

### Minimum Inhibitory Concentration (MIC) and Minimum Bactericidal Concentration (MBC) of PLA Against *L. innocua*

Minimum inhibitory concentration (MIC) and MBC of PLA against *L. innocua* ATCC 33090 were evaluated. The MIC was assessed at different pH values (4.5, 5.0, 5.5, 6.0, 6.5, and 7.0) using the macrobroth dilution method (Sorrentino et al., 2018) with some modifications. Trials were performed in BHI broth (Oxoid). At each pH value, tubes were inoculated with an overnight culture of *L. innocua* at final concentration of about 5 log CFU/mL. Serial twofold dilutions of PLA, containing from 30 to 0.23 mg/mL and from 20 to 0.15 mg/mL, were used. Tubes of BHI without PLA and inoculated with *L. innocua* as described above were used as control. Before inoculation, the pH of BHI was adjusted using HCl 1N or NaOH 1N. After 24 h, the turbidity of each tube was evaluated at 600 nm using a spectrophotometer (Bio-spectrometer basic, Eppendorf, Italy). 100 μL of the cell suspensions from the tubes without growth were sub-cultured on BHI agar (Oxoid) plates. MIC was defined as the lowest concentration of PLA at which bacteria failed to grow in liquid medium, but yet viable when 100 μL of the culture broth were plated on agar media. MBC was defined as the lowest concentration of PLA at which bacteria failed to grow in liquid medium, with a negative growth after incubation on agar media.

Using the same methods, the MIC and MBC of LA, GA, FA, and CA were assessed against *L. innocua* at pH 5.5.

For each acid, MIC and MBC values were expressed as mM of total acid and as mM of undissociated acid. The concentration of undissociated acid was calculated using the equation (1) which was derived from the Henderson and Hasselbalch equation:

(1) $$\begin{array}{c} {}[\mathrm{AcH}]\frac{\left[{\mathrm{Ac}}_{\mathrm{tot}}\right]}{{1+10}^{\left(pH-pKa\right)}} \end{array}$$

where:

AcH = undissociated acid concentration;
Ac_tot_ = total acid concentration;
pH = pH of medium used in MIC and MBC test;
pKa = dissociation constant of each acid.

### Antimicrobial Activity of PLA

The antimicrobial activity of PLA against *L. innocua* was evaluated by measuring the reduction in numbers (Log CFU/mL) over 24 h as described by Carson et al. (2002) with some modifications. Briefly, bacterial suspensions were prepared by centrifuging 150 mL of Mueller-Hinton Broth (MHB, Oxoid) overnight cultures (37°C for about 15 h) at 8000 rpm for 10 min at 4°C. The pellets were washed gently using MES buffer at pH 5.5 (Sigma-Aldrich), and then suspended in 150 mL of MES buffer in order to obtained a final cell density of about 10^5^ or 10^8^ CFU/mL. The *L. innocua* MES suspension was divided into three batches prepared as follows:

PLA_MIC: PLA was added to *L. innocua* suspension at the MIC concentration detected at pH 5.5;
PLA_MBC: PLA was added to *L. innocua* suspension at the MBC concentration detected at pH 5.5;
C: Control, cell suspension without PLA addition.

In addition, cells suspended in MES buffer were added with LA as follows:

LA_MIC: LA was added to *L. innocua* suspension at the MIC concentration detected at pH 5.5;
LA_PLA: LA was added at the same concentration of PLA used in the batch PLA_MIC to *L. innocua* suspension;

Finally, the same experiment was carried out using GA, FA and CA at MIC and MBC concentration.

For each test, the suspensions were adjusted at pH 5.5 after addition of acids.

The suspensions were incubated at 37°C and, at regular time intervals, aliquots of 1 mL were taken from the suspensions and then were serially diluted and plated on BHI agar (Oxoid). The plates were incubated at 37°C for 48 h before enumeration.

The survival data were modeled using the linear or the non-linear regression approach as reported by Succi et al. (2017a). For this purpose, the Geeraerd and Van Impe Inactivation Model Fitting Tool (GInaFiT) was used (Geeraerd et al., 2005).

### Bacterial Surface Charge: Zeta Potential

The zeta potential of bacterial suspensions containing PLA at the MBC concentration, in ultrapure water (pH 6.0), was determined using a Nano Zetasizer (Malvern Instruments, Ltd., United Kingdom). Cell suspensions, without acids, were used as controls. GA, CA, and FA at the MBC concentration were used as additional controls. The zeta potential was measured by applying an electric field across the bacterial suspensions.

### Loss of Cellular Content

The release of cellular content into supernatant was measured according to the method described by Rhayour et al. (2003). Cells from overnight cultures (15 mL) were collected by centrifugation (5500 rpm for 15 min), washed three times and suspended in MES buffer (pH 5.5). PLA, GA, CA, or FA at the MBC concentration were added to cell suspensions and incubated at 37°C for 5 h. Cell suspensions without phenolic compound were used as control. Then, 10 mL of each batch were taken and filtered through a 0.22 μm-pore-size filter (Carson et al., 2002). The concentration of the cellular content released was determined by UV absorption measurements of each filtrate using an UV-spectrophotometer (Bio-spectrometer basic) at 260 nm.

### Statistical Analysis

Statistical analysis was performed on three independent experiments through ANOVA followed by the Tuckey’s multiple comparison. For this purpose, the software GraphPad Prism version 6.0 was used. Statistical significance was attributed to *P-*values < 0.05. Statistical data were expressed as mean ± standard deviation.

## Results and Discussion

### MIC Values of PLA at Different pH

*Listeria innocua* was chosen to investigate the antilisterial action expressed by PLA, considering that this species is regarded as a non-pathogenic indicator for the presence of *L. monocytogenes* in foods and the high similarity between the two species (Rosimin et al., 2016; Tremonte et al., 2016b).

**FIGURE 1:**
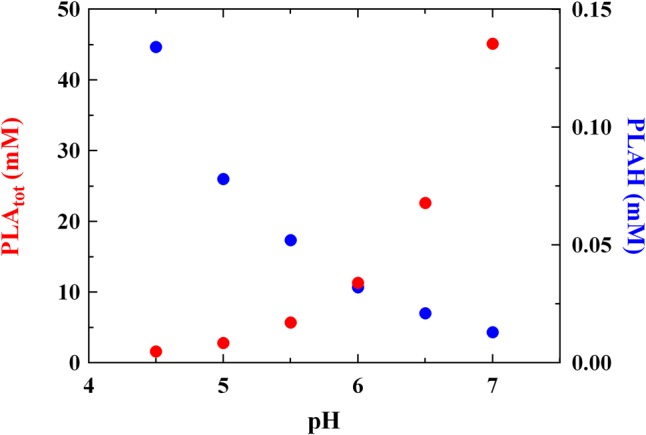
Minimum inhibitory concentration (MIC, mM) of total (PLA_tot_) and undissociated (PLAH) phenyllactic acid against *Listeria innocua* ATCC 33090 detected in BHI at different pH values.

The MIC levels of PLA detected at different pH values are reported in **Figure 1**. The results are in accordance with MICs already reported by Ning et al. (2017) for PLA *vs L. monocytogenes*. These Authors revealed a MIC value of about 1.25 mg/mL but no information regarding data relation between pH values and antimicrobial activity was given. In our study, a correlation between MIC and pH values was found (*r* = 0.90) and a significant decrease (*P* < 0.05) of the antilisterial activity due to PLA was detected at the highest pH values tested. In detail, the antilisterial action was pH-dependent, highlighting the typical activity of other weak acid preservatives and organic acids, such as LA. Moreover, at pH 7.0 the concentration of undissociated PLA (PLAH) was about 0.013 mM and the highest MIC value was observed (about 45 mM). Conversely, at pH 4.5 PLAH concentration was 0.134 mM, and the lowest MIC value (1.6 mM) was detected (**Figure 1**). Using intermediate pH values, that is, pH 5.0, 5.5, 6.0 and 6.5, PLAH concentration decreased (0.078, 0.052, 0.032, and 0.021 mM, respectively) and MIC values increased (2.8, 5.7, 11.3, and 22.6 mM, respectively). These data allows to claim that the PLA mode of action is related to its undissociated form (pKa 3.46) able to cross the microbial membrane. In fact, other studies already showed that the antimicrobial activity of some acids is ascribable to the ability of undissociated forms to freely cross the cytoplasmic membrane (Brul and Coote, 1999), inducing damages to the membrane permeability (Wang et al., 2015), thus justifying their long use as food additives and preservatives for inhibiting the microbial growth. Specifically, their inhibitory mechanism is attributable to the pH reduction (Wang et al., 2015), which induces protons release from undissociated molecules in the cytoplasm. This fact lead to a decrease of the intracellular pH, inhibiting essential microbial metabolic reactions (Olasupo et al., 2004).

These findings confirm what reported previously by Presser et al. (1997) and Coroller et al. (2005), which specified that the pH effect could not be considered in the same manner as temperature or wather activity, since pH changes in foods induce modifications in amounts of undissociated forms of organic acids linked to their specific pka. For this reason, it could not be expected that the MIC of a specific organic acid should be the same whatever the pH, because not only a change in hydrogen ion concentration occurs, but also considering that undissociated amounts of organic acids are pH-dependent.

Some Authors (Lavermicocca et al., 2003; Prema et al., 2010; Cortés-Zavaleta et al., 2014) reported higher MIC values for PLA tested against molds than that revealed in the present study. This fact suggests that PLA shows a higher effectiveness against bacteria, whereas for many years it was considered an antifungal metabolite (Lavermicocca et al., 2003; Vermeulen et al., 2006). Moreover, PLA amounts compatible with those observed in cultures of LAB (Rodríguez et al., 2012; Cortés-Zavaleta et al., 2014; Corsetti et al., 2015) had functional antilisterial activity, as showed by concentrations of 2.8 and 5.7 mM detected at pH 5.0 and 5.5 (corresponding to MIC values at the same pH, **Figure 1**), respectively.

### Effect of PLA and Lactic Acid on *L. innocua* Decay

In order to understand the PLA mode of action, the antilisterial effect produced by PLA at pH 5.5 was compared to the effect induced by LA at the same pH value. The antilisterial activity was evaluated at pH 5.5 since this pH value is closer to that characterizing high-risk foods associated with listeriosis, such as cheeses and fermented sausages (Di Luccia et al., 2016; Niro et al., 2017; Tremonte et al., 2017b). LA was chosen because its antimicrobial mechanism is well-known (Wang et al., 2015), and because it represents the main metabolic product of LAB, also able to produce PLA.

In **Table 1**, MIC and MBC of LA and PLA detected at pH 5.5 against *L. innocua* ATCC 33090 are reported. Data evidenced a significant difference (*P* < 0.05) between PLA and LA for both MIC and MBC values. Specifically, when LA (Actot) was used, a concentration 40-fold higher than PLA (Actot) was needed to produce an inhibitory action *vs L. innocua*. This difference became even more evident when the undissociated (AcH) form was tested: in this case, the MIC of LA was about 95-fold higher than that showed by PLA. A similar behavior was observed in the assessment of MBC.

**Table 1:** MIC and MBC values (mM) of total (Ac_tot_) and undissociated (AcH) phenyllactic acid (PLA), gallic acid (GA), caffeic acid (CA), ferulic acid (FA), and lactic acid (LA) against *L. innocua* ATCC 33090 grown in BHI broth at pH 5.5.

Samples		PLA	LA	GA	CA	FA
MIC (mM)	Ac_tot_	5.7 ± 0.7a	222.0 ± 2.1e	7.3 ± 0.5b	10.4 ± 1.1c	12.9 ± 0.6d
	AcH	0.052 ± 0.006a	4.97 ± 0.05e	0.54 ± 0.04b	0.80 ± 0.09c	1.17 ± 0.05d
MBC (mM)	Ac_tot_	11.3 ± 1.3a	577.3 ± 3.2e	14.8 ± 0.8b	28.4 ± 0.7c	25.9 ± 0.4d
	AcH	0.102 ± 0.012a	12.93 ± 0.07e	1.09 ± 0.06b	2.18 ± 0.05c	2.35 ± 0.04d

*Mean ± standard deviation of three independent experiments. Means in the same row with different letters are significantly different (*P* < 0.05).*

**FIGURE 2:**
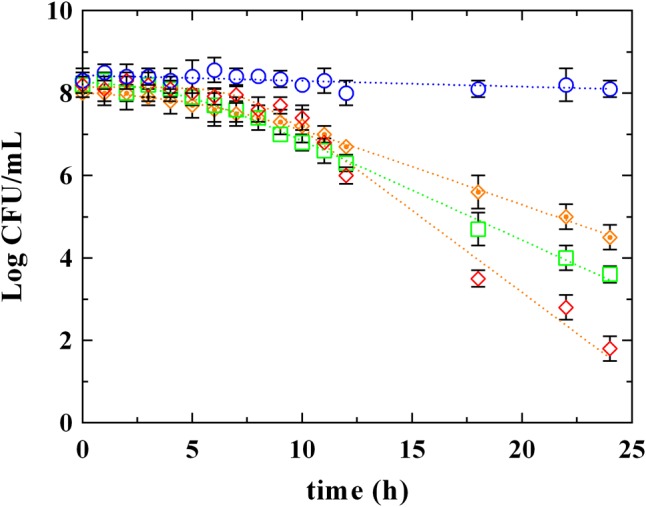
Survival of *L. innocua* ATCC 33090 in (∘) MES buffer (C), or in MES buffer containing: (♢), phenyllactic acid at MIC concentration detected at pH 5.5 (PLA_MIC); (□), lactic acid (LA) at MIC concentration detected at pH 5.5 (LA_MIC) or (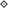), LA at the same concentration of MIC of PLA (LA_PLA). Symbols represent the mean values with standard deviation of three independent experiments and the curves represent the survival models obtained with GInaFiT software.

The survival kinetics of *L. innocua* (**Figure 2**) were evaluated in MES buffer added with PLA and LA at the respective MIC concentrations detected at pH 5.5 (PLA_MIC and LA_MIC, respectively). For comparative purposes, kinetic curves were also evaluated in the same medium added with LA used at the same concentration of PLA (LA_PLA) and without antimicrobial addition (control condition, C). The corresponding survival kinetic parameters (shoulder, maximum death rate and 4D values) are reported in Supplementary Table S1. As expected, substantially constant loads of *L. innocua* were detected in the control condition during the entire observation period, while a significant decrease in counts (*P* < 0.05) was observed in presence of both PLA and LA. In detail, the survival of *L. innocua* in C was described by a Log-linear model, whereas the mathematical function that better described the survival of *L. innocua* in presence of LA or PLA was a Log-linear model with shoulder. Moreover, significant differences were also detected between the kinetic curves obtained in presence of PLA or LA. In fact, a K_max_ value of -0.92 h^-1^ was detected for PLA, and it was significantly higher than that obtained with LA_MIC (-0.56 h^-1^) and LA_PLA (0.43 h^-1^). A higher resistance (shoulder, S1) of *L. innocua* was appreciated in presence of LA_MIC and LA_PLA (4.2 and 5.5 h, respectively) respect to that observed in presence of PLA_MIC (7.5 h).

Based on these results, it is possible to assert that the antimicrobial action of PLA is substantially different from LA. As also reported by Ning et al. (2017), the strong antimicrobial effect produced by PLA could be due to its amphiphilic properties, which allow an interaction with lipids and proteins of cytoplasmic membrane and, probably, an intercalation in the bacterial DNA. For this reason, a comparison between PLA and the most studied phenolic acids (hydroxybenzoic and hydroxycinnamic ones) was carried out.

### Effect of Phenolic Compounds on *L. innocua* Decay

To understand the antimicrobial effect of PLA on *L. innocua*, further experiments were conducted using three widely studied phenolic compounds: gallic (GA), caffeic (CA), and ferulic (FA) acids (**Table 1**). The mechanism of action expressed by the hydroxybenzoic (GA) and hydroxycinnamic (FA and CA) acids against different undesirable microorganisms, including both Gram negative and Gram positive bacteria, was already described by Gutiérrez-Larraínzar et al. (2012), Borges et al. (2013), Fernàndez-Àlvarez et al. (2014), Chen et al. (2017), and Sorrentino et al. (2018). On the basis of the results reported by these Authors, hydroxybenzoic and hydroxycinnamic acids seem to led to irreversible changes in membrane properties (charge, intra and extracellular permeability), to a decrease of negative surface charge, as well as to rupture or pore formation in the cell membranes with consequent loss of intracellular constituents. The MIC and MBC values of phenolic acids against *L. innocua* were evaluated in the same conditions described above, and they were always higher than those registered with the use of PLA, highlighting a greater effectiveness of the latter in both dissociated (Actot) and undissociated (AcH) forms.

**FIGURE 3:**
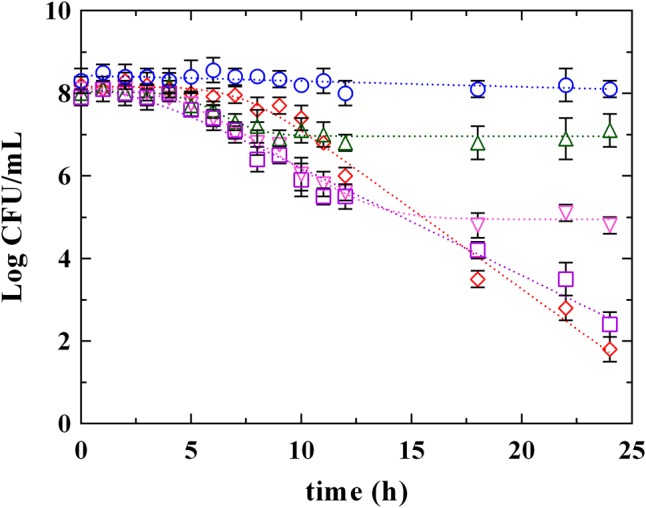
Survival of *L. innocua* ATCC 33090 in (∘) MSE buffer (C), or in MSE buffer containing: (□), gallic acid (GA_MIC); (△), ferulic acid (FA_MIC); (▽), caffeic acid (CA_MIC) or (♢), phenyllactic acid (PLA_MIC) at MIC concentration detected at pH 5.5. Symbols represent the mean values with standard deviation of three independent experiments and the curves represent the survival models obtained with GInaFiT software.

**FIGURE 4:**
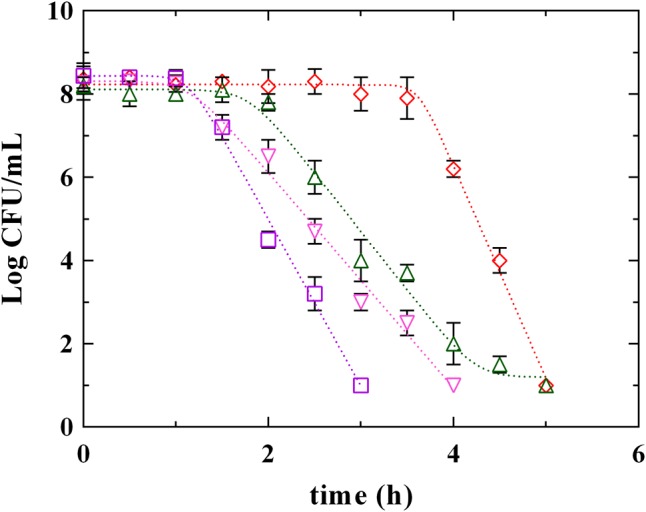
Survival of *L. innocua* ATCC 33090 in MES buffer containing: (□), gallic acid (GA_MBC); (△), ferulic acid (FA_MBC); (▽), caffeic acid (CA_MBC) or (♢), phenyllactic acid (PLA_MBC) at MBC concentration detected at pH 5.5. Symbols represent the mean values with standard deviation of three independent experiments and the curves represent the survival models obtained with GInaFiT software.

**Figures 3, 4** report the survival kinetic curves of *L. innocua* in presence of PLA and phenolic acids (GA, FA, and CA) used at MIC or MBC concentrations, respectively. The corresponding kinetic parameters are reported in Supplementary Tables S2, S3, respectively.

As reported above (Supplementary Table S1), a Log-linear model and a Log-linear model with shoulder described the behavior of *L. innocua* in control conditions (C) and in presence of PLA_MIC, respectively. Different models described instead the survival of *L. innocua* in presence of phenolic acids. Specifically, a Log-linear model was individuated in the case of GA_MIC and Log-linear models with shoulder and tail were detected in presence of FA_MIC and CA_MIC (Supplementary Table S2). The highest inhibition was appreciated in presence of PLA_MIC and GA_MIC (**Figure 3** and Supplementary Table S2) which produced a strong inhibition, showing similar (*P* > 0.05) 4D values (17.5 and 18.0 h, respectively). In contrast, the effects produced by hydroxycinnamic acids CA and FA were less pronounced respect to previous ones. However, significant differences between PLA_MIC and GA_MIC were observed. In fact, the shoulder length was estimated in 7.3 h in presence of PLA_MIC and in 2.5 h in presence of GA_MIC.

Significant differences among PLA and phenolic compounds were detected also when they were used at MBC concentrations (**Figure 4** and Supplementary Table S3), although in all cases a complete decay of *L. innocua* was observed within 5 h. In fact, a Log-linear model with shoulder and tail described the survival of *L. innocua* in presence of FA_MBC while Log-linear models with shoulder were individuated in presence of PLA_MBC, CA_MBC, and GA_MBC. Specifically, *L. innocua* in presence of PLA showed the highest values of 4D and of shoulder length, highlighting, once again, a different mechanism of action respect to other antimicrobial organic compounds. The data set generated from both MIC an MBC highlighted that PLA showed differences respect to both hydroxybenzoic (GA) and hydroxycinnamic (FA and CA) acids, suggesting that PLA produces an antilisterial activity through a specific mechanism which is different from those usually observed for other phenolic compounds. Actually, the effects of phenolic acids on the bacterial cells stability and survival were not yet completely individuated (Succi et al., 2017b), even if several studies focused on the antimicrobial activity of these compounds and demonstrated that they involve numerous mechanisms of action, such as permeability destabilization and enzymes inhibition through a non-specific interaction (Ota et al., 2011; Hayrapetyan et al., 2012; Borges et al., 2013). So it is conceivable that PLA acts similarly, using more than one of these pathways, but differently from other phenolic compounds.

### Effect of Phenolic Compounds on Surface Charge and Loss of Cellular Content

**FIGURE 5:**
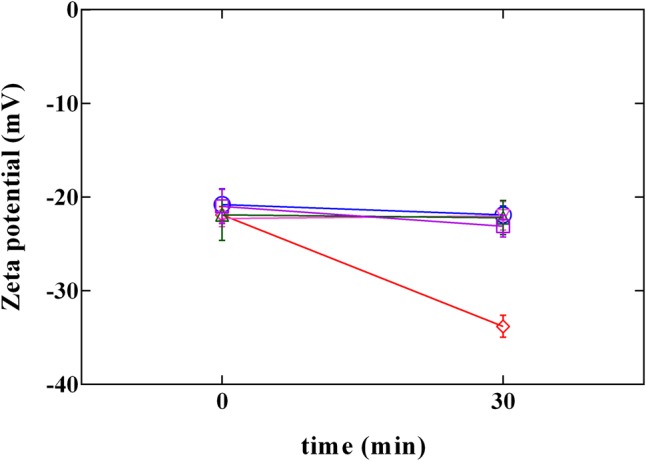
Zeta potential of *L. innocua* ATCC 33090 in (∘) ultrapure water (C), or in ultrapure water containing: (□), gallic acid (GA_MBC); (△), ferulic acid (FA_MBC); (▽), caffeic acid (CA_MBC) or (♢), phenyllactic acid (PLA_MBC) at MBC concentration detected at pH 5.5.

Cell membrane has been proposed as one of the most important target of phenolic compounds (Hayrapetyan et al., 2012; Borges et al., 2013). Therefore, information on surface charge change or on loss of cellular content could provide important clarification regarding antimicrobial mechanisms. Due to the presence of anionic groups (e.g., carboxyl and phosphate), the surface charge of bacterial cells is generally negative (Wang et al., 2015). Data obtained by the measurement of zeta potential (**Figure 5**) showed the charge change in *L. innocua* after exposure to PLA and phenolic compounds. Zeta potential measurements demonstrated that, after exposure, cells became more (*P* < 0.05) negatively charged when exposed to PLA, while in presence of phenolic compounds (GA, CA, and FA) no variation in charge was detected. This result confirm data reported in literature (Borges et al., 2013), which attributed no effect to hydroxycinnamic (FA) and hydroxybenzoic (GA) acids in Gram-positive charge changes. In fact, so far, change in charge surface produced by phenolic acids was observed only in Gram negative bacteria. On the other hand, results obtained by PLA allow to assume that the antilisterial action of PLA is associated with the affinity to the cell surface, and the interaction PLA-cell surface could contribute to the damage of cellular structures.

**FIGURE 6:**
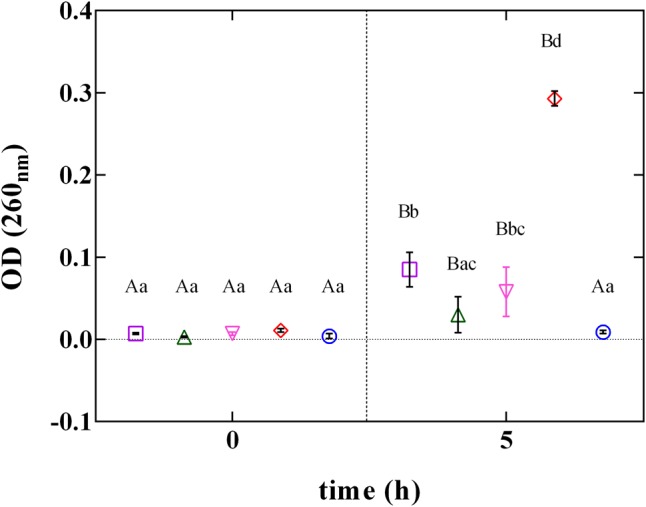
Cellular loss content of *L. innocua* ATCC 33090 in (∘) ultrapure water (C), or in ultrapure water containing: (□), gallic acid (GA_MBC); (△), ferulic acid (FA_MBC); (▽), caffeic acid (CA_MBC) or (♢), phenyllactic acid (PLA_MBC) at MBC concentration detected at pH 5.5.

The rupture of cellular structures was also supported by the results of the cellular content loss (**Figure 6**). The release of cell constituents was determined by the measurement of the absorbance at 260 nm of the filtrates of *L. innocua* cultures. The treatment with PLA at MBC concentration induced a significant (*P* < 0.05) increase in cellular content release. Contrarily, when *L. innocua* cultures were exposed to the other phenolic acids (GA, FA, and CA) used at the MBC concentration, only a weak variation in OD values was detected. This finding, integrated with results from zeta potential, demonstrates that at pH 5.5 PLA strongly interacts with the surface of *L. innocua* cells, promoting membrane damage, release of intracellular content and the consequent cell death.

These results might help to explain the differences in the antilisterial mechanism of phenolic compounds. In fact, hydroxybenzoic and hidroxycinnamic acids seem to induce an alteration in membrane permeability without causing its rupture, whereas PLA, having the main targets in the cellular surface and in the cytoplasmic membrane, leads to a severe rupture of the cellular structures.

## Conclusion

The results obtained through the present study open new horizons in the understanding of PLA antilisterial mechanism. The relation between pH values and antilisterial activity of PLA was clarified, showing that the antimicrobial action of PLA was substantially different from weak acids, such as LA. This difference could be ascribable to the chemical structure of phenolic acids (hydrophobic group-benzene ring and hydrophilic group-carboxy). Nevertheless, differences between PLA and well-known phenolic acids (hydroxybenzoic and hidroxycinnamic acids) were detected when considering the death kinetic parameters. These items suggest that PLA produces an antagonistic activity against *L. innocua* through specific mechanisms, which are different from those of LA and phenolic compounds. It is possible to hypothesize that the PLA antilisterial action is also associated with the affinity with the cell surface, and that the interaction PLA-cell surface could contribute to the damage of cellular structures. The specific antilisterial mechanism could explain the MIC value *vs L. innocua* which appear lower than that of the other tested compounds (LA, phenolic acids) and compatible with the maximum PLA production generally observed in cultures of LAB.

## Author Contributions

ES: design of the work and drafting the work. PT: design of the work, analysis and interpretation of the anti-microbial data, and drafting the work. MS: interpretation of the data, drafting the work, and revising it critically. MI: involved in experimental designing. GP: analysis and interpretation of data and revising it critically. SL: analysis and interpretation of antilisterial data. MSt: analysis of zeta potential a and cellular loss content. RC: conception of the work, agreement to be accountable for all aspects of the work in ensuring that questions related to the accuracy or integrity of any part of the work are appropriately investigated and resolved.

## Conflict of Interest Statement

The authors declare that the research was conducted in the absence of any commercial or financial relationships that could be construed as a potential conflict of interest.
