# Supplementary material for: Detection of Antilisterial Activity of 3-Phenyllactic Acid Using Listeria innocua as a Model
Source: Front Microbiol. 2018 Jun 26;9:1373. doi: 10.3389/fmicb.2018.01373 (PMC6028618; doi:10.3389/fmicb.2018.01373)
Supplement: Supplementary file 1 [file Table_1.pdf]

Supplementary Material

Detection of antilisterial activity of 3-phenyllactic acid using *Listeria innocua* as a model

Elena Sorrentino*, Patrizio Tremonte, Mariantonietta Succi, Massimo Iorizzo, Gianfranco Pannella, Silvia Jane Lombardi, Marina Sturchio, Raffaele Coppola

*** Correspondence:** Elena Sorrentino: sorrentino@unimol.it

Supplementary table

Table S1 Survival kinetic parameters estimated on *L. innocua* ATCC 33090 after exposure to phenyllactic acid at MIC concentration detected at pH 5.5 (PLA_MIC), lactic acid at MIC concentration (LA_MIC), lactic acid at same concentration of MIC of PLA (LA_PLA) or without preservative agents (C).

Samples	Models	$Log(N_0)$ (Log CFU/mL)	Sl (h)	K_{max} (h^{-1})	4D (h)	RMSE	adj-R^2
C	Log-linear	8.4 ± 0.2a	-	0.03 ± 0.02a	>24	0.116	0.385
PLA_MIC	Log-linear + S	8.2 ± 0.2a	7.5 ± 0.4a	0.92 ± 0.07b	17.5 ± 0.9a	0.241	0.988
LA_MIC	Log-linear + S	8.3 ± 0.3a	4.2 ± 0.3b	0.56 ± 0.05c	20.9 ± 0.7b	0.111	0.995
LA_PLA	Log-linear + S	8.2 ± 0.2a	5.5 ± 0.4c	0.43 ± 0.05d	>24	0.126	0.935

Mean ± standard deviation of three independent experiments. Means in the same column with different letters are significantly different ($P < 0.05$). Log-linear + S, Log-linear model with shoulder; N_0, Initial inoculum concentration; Sl, Shoulder length; K_{max}, First order inactivation rate constant; 4D, Logcycles of reduction; RMSE, Root mean sum of squared error; adj-R^2, R-square adjusted coefficient of determination.
