# Supplementary material for: Detection of Antilisterial Activity of 3-Phenyllactic Acid Using Listeria innocua as a Model
Source: Front Microbiol. 2018 Jun 26;9:1373. doi: 10.3389/fmicb.2018.01373 (PMC6028618; doi:10.3389/fmicb.2018.01373)
Supplement: Supplementary file 2 [file Table_2.pdf]

Supplementary Material

Detection of antilisterial activity of 3-phenyllactic acid using *Listeria innocua* as a model

Elena Sorrentino*, Patrizio Tremonte, Mariantonietta Succi, Massimo Iorizzo, Gianfranco Pannella, Silvia Jane Lombardi, Marina Sturchio, Raffaele Coppola

*** Correspondence:** Elena Sorrentino: sorrentino@unimol.it

Supplementary table

Table S2 Survival kinetic parameters estimated on *L. innocua* ATCC 33090 after exposure to gallic acid (GA), cinnamic acid (CA), ferulic acid (FA), phenyllactic acid (PLA) at MIC concentration detected at pH 5.5 or without preservative agents (C).

Samples	Models	$Log(N_0)$ (Log CFU/mL)	Log(N_res) (Log CFU/mL)	Sl (h)	K_{max} (h^{-1})	4D (h)	RMSE	adj-R^2
C	Log-linear	8.4 ± 0.2a	-	-	0.03 ± 0.02a	>24	0.116	0.385
PLA_MIC	Log-linear + S	8.2 ± 0.2a	-	7.5 ± 0.4a	0.92 ± 0.07b	17.5 ± 0.9a	0.241	0.988
GA_MIC	Log-linear + S	8.2 ± 0.1a	-	2.5 ± 0.5b	0.60 ± 0.11c	18.0 ± 1.1a	0.264	0.978
FA_MIC	Log-linear + S + T	8.0 ± 0.3a	7.0 ± 0.3a	5.2 ± 0.1c	1.15 ± 0.05d	>24	0.126	0.935
CA_MIC	Log-linear + S + T	8.1 ± 0.2a	5.0 ± 0.1b	4.2 ± 0.3d	0.79 ± 0.06b	>24	0.119	0.989

Mean ± standard deviation of three independent experiments. Means in the same column with different letters are significantly different ($P < 0.05$). Log-linear + S, Log-linear model with shoulder; Log-linear + S + T, Log-linear model with shoulder and tail; N_0, Initial inoculum concentration; N_res, Starting point of tail; Sl, Shoulder length; K_{max}, First order inactivation rate constant; 4D, Logcycles of reduction; RMSE, Root mean sum of squared error; adj-R^2, R-square adjusted coefficient of determination.
