# Supplementary material for: Detection of Antilisterial Activity of 3-Phenyllactic Acid Using Listeria innocua as a Model
Source: Front Microbiol. 2018 Jun 26;9:1373. doi: 10.3389/fmicb.2018.01373 (PMC6028618; doi:10.3389/fmicb.2018.01373)
Supplement: Supplementary file 3 [file Table_3.pdf]

Supplementary Material

Detection of antilisterial activity of 3-phenyllactic acid using *Listeria innocua* as a model

Elena Sorrentino*, Patrizio Tremonte, Mariantonietta Succi, Massimo Iorizzo, Gianfranco Pannella, Silvia Jane Lombardi, Marina Sturchio, Raffaele Coppola

*** Correspondence:** Elena Sorrentino: sorrentino@unimol.it

Supplementary table

Table S3 Survival kinetic parameters estimated on *L. innocua* ATCC 33090 after exposure to gallic acid (GA), cinnamic acid (CA), ferulic acid (FA) or phenyllactic acid (PLA) at MBC concentration detected at pH 5.5.

Samples	Models	Log(N_0) (Log CFU/mL)	Log(N_res) (Log CFU/mL)	Sl (h)	K_{max} (h^{-1})	4D (h)	RMSE	adj-R^2
PLA_MBC	Log-linear + S	8.2 ± 0.1a	-	3.6 ± 0.7a	11.8 ± 0.4a	4.5 ± 0.5a	0.185	0.994
GA_MBC	Log-linear + S	8.4 ± 0.2a	-	1.1 ± 0.3b	9.2 ± 1.1b	2.2 ± 0.4b	0.291	0.991
FA_MBC	Log-linear + S + T	8.1 ± 0.2a	1.2 ± 0.2	1.8 ± 0.2c	6.5 ± 0.7c	3.3 ± 0.2c	0.366	0.984
CA_MBC	Log-linear + S	8.3 ± 0.3a	-	1.2 ± 0.3b	6.5 ± 0.8c	2.7 ± 0.4b	0.303	0.988

Mean ± standard deviation of three independent experiments. Means in the same column with different letters are significantly different ($P < 0.05$). Log-linear + S, Log-linear model with shoulder; Log-linear + S + T, Log-linear model with shoulder and tail; N_0, Initial inoculum concentration; N_res, Starting point of tail; Sl, Shoulder length; K_{max}, First order inactivation rate constant; 4D, Logcycles of reduction; RMSE, Root mean sum of squared error; adj-R^2, R-square adjusted coefficient of determination.
